# Unlocking the Potential of Pepper Plants under Salt Stress: Mycorrhizal Effects on Physiological Parameters Related to Plant Growth and Gas Exchange across Tolerant and Sensitive Genotypes

**DOI:** 10.3390/plants13101380

**Published:** 2024-05-16

**Authors:** Ozlem Altuntas, Hayriye Yildiz Dasgan, Yelderem Akhoundnejad, Yahya Nas

**Affiliations:** 1 Department of Horticulture, Faculty of Agriculture, University of Malatya Turgut Ozal, 44900 Malatya, Turkey; 2 Department of Horticulture, Faculty of Agriculture, University of Çukurova, 01330 Adana, Turkey; dasgan@cu.edu.tr; 3 Department of Horticulture, Faculty of Agriculture, University of Sirnak, 73000 Sirnak, Turkey; yakhoundnejad@sirnak.edu.tr (Y.A.); yahya.nas@sirnak.edu.tr (Y.N.)

**Keywords:** Arbuscular mycorrhizal fungi (AMFs), *Capsicum annuum* L., NaCl stress, photosynthesis, stomatal conductance, leaf water potential, plant growth

## Abstract

Agriculture is confronted with the challenge of ensuring global food security, yet the rapid expansion of salinity stress undoubtedly restricts plant productivity in cultivable areas, posing a significant threat to crop yields. Arbuscular mycorrhizal fungi (AMFs) have emerged as a biological tool for enhancing plant salt stress tolerance. To utilize this biological tool, this study evaluated the response in growth and physiological parameters of tolerant (Karaisali) and sensitive (Demre) pepper genotypes. The experiment involved mycorrhizal-treated (*Glomus clarium*) and non-mycorrhizal (control) plants of both the tolerant and sensitive pepper genotypes. The plants were subjected to two salt doses: 75 and 150 mM. The plant growth and physiological parameters were measured at 40 days after transplanting. The mycorrhizal activity was found to be significantly more effective in the sensitive genotype. We found notable differences in mycorrhizal activity between the pepper genotypes under salt stress conditions, with the sensitive genotype “Demre” showing greater responsiveness to mycorrhizal association compared with the “Karaisali” variety. Under both moderate (75 mM NaCl) and higher salt stress levels (150 mM NaCl), both the “Karaisali” and “Demre” varieties exhibited substantial increases in their shoot dry weights. However, these increases were consistently higher in the “Demre” plants. Moreover, the AMFs demonstrated significant enhancements in photosynthesis rates under both moderate and high salinity levels in both genotypes. Overall, our findings suggest that AMFs play a crucial role in improving plant growth, water status, and photosynthesis characteristics, particularly in salt-sensitive pepper genotypes, under moderate-to-high salinity levels. In conclusion, the plant growth, water status, and photosynthesis characteristics of the salt-sensitive pepper genotype were significantly improved by AMFs at medium and high salinity levels, such as 75 mM and 150 mM NaCl, respectively.

## 1. Introduction

Mycorrhizal symbiosis is the most common symbiosis in nature. This symbiosis is an evolutionarily primitive microbiota and, according to the fossil record and molecular data, occurred with the first terrestrial plants [1,2]. Paleobotanical and molecular sequence data indicate that the first land plants established associations with Glomalean fungi in the Glomeromycota approximately 460 million years ago [3]. The symbiotic relationship formed by fungi with plant roots functions like an extended root system. In this symbiotic relationship, fungi help the plant in the intake of water and nutrients with their hyphae, while the fungi receive carbohydrates, the product of photosynthesis, from the host plant [4]. The most important type of mycorrhiza for vegetable growers is the arbuscular mycorrhizal symbiosis. Employing mycorrhizal symbiosis is an effective strategy for enhancing plant tolerance to salt stress [5]. The outer hyphae of mycorrhiza can form a massive mycelium network in the soil, which can expand the absorption range of the root system [6], helping host plants absorb low-concentration nutrients and enhancing the exchange of substances between AMFs in the soil and plants [7].

Numerous ongoing research studies focus on AMFs and their symbiotic association with plants, aiming to elucidate their essential roles in plant growth, plant protection, and soil quality improvement. The reports of these studies provided much evidence that association with AMFs facilitates better nutrient uptake, enhancing plant growth under harsh soil (salinity) and climate conditions (drought). It has been reported by many researchers that AMFs promote plant growth and resistance to salt stress. Many authors have reported that mycorrhizal fungi increase tolerance to salt stress through various mechanisms, among which increasing nutrient uptake, producing plant growth hormones, and improving rhizospheric and soil conditions are to be mentioned [8,9,10].

The abiotic stresses resulting from climate change, driven by global warming, pose significant challenges to crop production worldwide. The costs of dealing with abiotic stresses are potentially enormous, and robust, affordable, environmentally friendly approaches are needed to reduce the negative effects of these on plants. Effective use of mycorrhizae that provides tolerance to stress is of critical importance to ensuring sustainable agriculture in everchanging environmental conditions. The association of AMFs enhances the ability of vegetable plants to withstand various types of stress to some extent. Mycorrhizae assist host plants to adjust the photosynthesis process through leaving CO_2_ regulation and maintaining the leaf water content during the high-pressure osmotic potential [11]. This phenomenon is provided by performing mycorrhizal spores when it germinates and penetrates the plant root hairs en route to establishing symbioses. An essential harness forms in between the rhizosphere and plant leaves when mycorrhizal hyphae uptake water from the soil, which non-mycorrhizal plants are unable to use through high-salt concentrations. It was found that the percentage variation in the growth rate (i.e., root and shoot lengths and root and shoot dry weights) and nutrient uptake in plants under drought and different levels of salinity stress was directly proportional to the percentage of mycorrhization [12]. AMF symbiosis can affect the stomatal behavior and photosynthesis of host leaves and is confirmed to increase both the transpiration and photosynthetic rates along with the chlorophyll concentration [13]. It has been observed that inoculation with AM fungus alone or in combination with Rhizobium brought a significant increase in the chlorophyll “a”, chlorophyll “b”, and total chlorophyll content in ground nut, thereby increasing the rate of photosynthesis.

While Turkey boasts fertile land suitable for a variety of crops and horticulture in the Middle East, over time, its soil has experienced an accumulation of many ions and elements. This gradual buildup has caused a significant shift in soil composition toward higher salinity concentrations. Soil beneficial microorganisms such as AMFs are among the most promising elements to encourage growth of crops under this compelling circumstance. For several centuries, pepper (*Capsicum annuum* L.) has been one of the most important field and greenhouse crops in the Mediterranean region due to its economic profit. In particular, greenhouse cultivation demands higher levels of fertilization compared with field cultivation. However, the salt from these fertilizers does not leach deep into the soil with rainfall, necessitating measures to mitigate salt stress during cultivation. Since they establish associations with roots, mycorrhizae-infected plants are able to uptake more nutrients and water. Consequently, mycorrhizae treatments have great potential to maintain ion regulation and thus hinder yield loss under salt stress conditions.

Our hypothesis seeks to investigate the impact of mycorrhizae on the plant growth and physiological parameters in salt-sensitive genotypes, particularly in mitigating salt stress damage. While using salt-resistant genotypes is a feasible strategy for saline environments, harnessing beneficial microorganisms like mycorrhizae offers a more rational approach when cultivating productive and high-quality salt-sensitive varieties. The objective of our study is to evaluate the physiological support provided by mycorrhizae when growing salt-sensitive varieties in saline conditions.

## 2. Materials and Methods

### 2.1. Plant and Mycorrhizal Inoculation and Experimental Conditions

**Figure 1:**
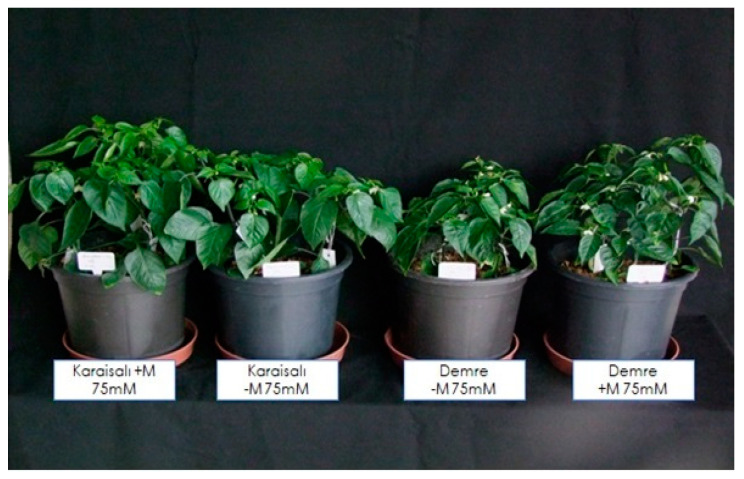

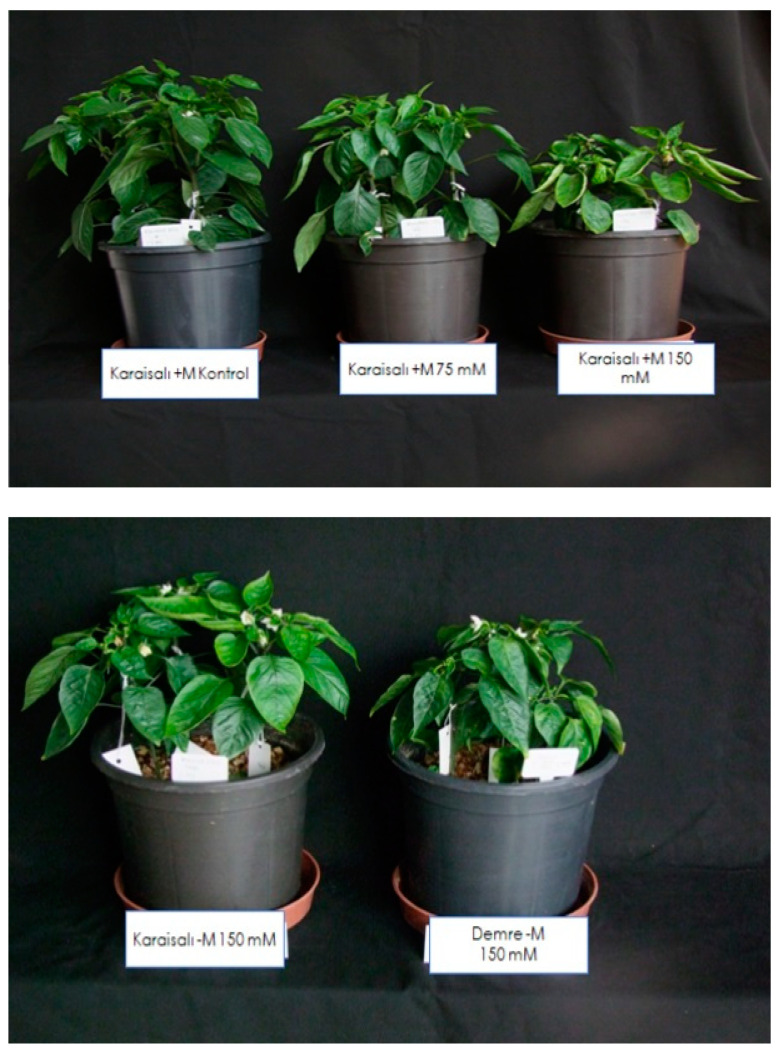
Responses of pepper plants to 75 mM and 150 mM salt stresses and AMF inoculation after 40 days of seedling transplanting and 26 days under salinity.

The experiment was conducted in the growth chamber of Cukurova University’s Department of Horticulture in the Faculty of Agriculture. To investigate the alleviation of harmful effects of salt stress on sensitive pepper plants through mycorrhiza inoculation, a pot experiment was designed with both non-mycorrhizal and mycorrhizal treatments. The salt-tolerant (Karaisali) and salt-sensitive (Demre) local pepper genotypes identified in previous studies were used [14]. The plants were treated with three different salt dosages of 0 (control), 75 mM, and 150 mM NaCl. Seedlings of both pepper varieties with the true leaf stage were inoculated with *Glomus clarum*, and a non-inoculated set was used to be left as the control group. The climatic conditions inside the growth chamber were 22 ± 2 °C during the day and 18 ± 2 °C at night, with a 16 h photoperiod and 60% relative humidity. The amount of photosynthetically active radiation received by the upper plant surfaces was 300 mol m^−2^ s^−1^. Four-liter pots filled with vermiculite were utilized for plant cultivation. Three pepper seedlings were transplanted into each pot. The experiment was designed based on a randomized block design with three replicates. The mycorrhizal treatments involved inoculating each plant with 1000 AMF spores during the seedling transplanting process. The nutrient solution used for plant nutrition was as follows (M): Ca(NO_3_)_2_4H_2_O, 3.0 × 10^−3^; K_2_SO_4_, 0.90 × 10^−3^; MgSO_4_7H_2_O, 1.0 × 10^−3^; KH_2_PO_4_, 0.2 × 10^−3^; H_3_BO_3_, 1.0 × 10^−5^; 10^−4^ M Fe EDTA, MnSO_4_H_2_O, 1.0 × 10^−6^; CuSO_4_5H_2_O, 1.0 × 10^−7^; (NH)_6_Mo_7_O_24_4H_2_O, 1.0 × 10^−8^; ZnSO_4_7H_2_O, 1 × 10^−6^. To enhance the effectiveness of the mycorrhizae, the nutrient solution was diluted by 50% to fulfill the nutrient requirements for plant nutrition. The salinity treatments were imposed upon the plants 14 days after transplanting the seedlings. Salt concentrations of 75 mM and 150 mM were gradually reached over 3 days. The plants were exposed to salt stress for 26 days. The morphological and physiological features were measured 40 days after transplanting (Figure 1).

### 2.2. Determinatione of Plant Physiological Parameters

Leaf water potential (MPa): This was measured by the pressure chamber with a Plant Water Status Console mark and 3005-1412 model instrument (Soil Moisture Equipment Corp., Goleta, CA, USA) [15]. Measurements were performed with the third leaf from the tips of the plants.

Osmotic potential (MPa): This was determined according to the process laid out by Kuçukkomurcu [16], where 1 g of fresh leaves from the fourth leaves of the plants was weighed and homogenized with 19 mL of distilled water. The homogenized leaf samples were kept at −20 °C. The homogenized samples were passed through 0.45 µm precision filters. These samples were measured with a freezing point osmometer (Gonotec Osmomat 3000, ELITechGroup Inc., Logan Utah, UT, USA). The osmotic potential was calculated according to the Van’t Hoff equation [17].

Photosynthetic rate, stomatal conductance, and transpiration rate: The youngest fully expanded leaves (from the fifth leaves from the tops of the pepper plants) of individual plants were used for gas exchange measurements. The gas exchange characteristics were measured during the 16 h photoperiod using an LICOR LI-6400 portable photosynthesis system (LI-COR, Inc., Lincoln, NE, USA) (block temperature: 25 °C; CO_2_ reference: 360 µmol CO_2_ mol^−1^; PAR: 500 µmol m^−2^s^−1^; flow rate: 300 µmol s^−1^) [18].

Membrane injury index (%) (ion leakage): Tissue ion (electrolyte) leakage was measured as an indicator of the ability of the cellular membranes to maintain integrity or recover from imposed stresses [19]. Electrolyte leakage was measured by taking five leaf discs from the second top leaf and placing them in tubes containing 10 mL of DI water [20]. After incubation for 5 h at 25 °C, the conductivity of the solution was measured using a (WTW and model Cond3110) portable conductivity meter. The tubes were then heated at 100 °C for 10 min, and the conductivity was remeasured (considered the total cellular electrolytes).

The injury index was estimated with the following formula:

I = (1 − (1 − T1/T2)/(1 − C1/C2)) × 100

where T1 and T2 are the initial and second measurements of conductivity, respectively, and C1 and C2 are the respective values of the controls [20].

Chlorophyll SPAD measurements: A portable chlorophyll meter (SPAD–502, Konica Minolta Sensing, Inc., Tokyo, Japan) was used to measure the leaf greenness of the pepper plants. For each plant, measurements were taken at four locations on each leaf, with three on each side of the midrib on all fully expanded leaves, and then averaged [20].

Shoot and root parameters: Three plants from each replicate were harvested, and the shoot dry weights and root dry weights were determined. The plant material for the dry weight was dried at 70 °C for two days [15].

Leaf Area: Three plants from each replicate were harvested and measured using a LICOR 3100 (LI-COR, Inc., Lincoln, NE, USA) leaf area meter [15].

### 2.3. Statistical Analysis

The data were subjected to factorial analysis of variance using the Statistical Package for Social Sciences (SPSS version 20), and the means were separated by Duncan’s post hoc tests at the 5% level (*p* < 0.05).

## 3. Results

### 3.1. Effects of AMFs under Salt Stress on Pepper’s Plant Growth Parameters

The “Karaisali” variety achieved the higher shoot biomass (almost up to double the value) compared with that of the “Demre” variety without mycorrhizal inoculation 40 days after transplanting at a 150 mM salinity level (Table 1). The root dry weight also showed a similar pattern in the “Karaisali” and “Demre” varieties when the plants were exposed to a 150 mM salinity concentration 40 days after transplanting. Our observations indicate that the mycorrhization network provided a beneficial relationship with the “Karaisali” plant rather than the “Demre” plant in order keep growing through salinity situations and a higher osmotic pressure. The symbiotic relationship between plants and mycorrhizal fungi may enhance plant growth, as evidenced by the significant increase in the shoot dry weight observed in the “Karaisali” variety. This feature was also observed for the root dry weight compared with the “Demre” plant. The “Demre” plant did not become more productive as the salinity level increased during its growth time. However, the root dry weight clearly confirmed the mycorrhizal symbioses with an enhanced dry mass 40 days after transplanting the “Karaisali” plant. The dry mass of the “Karaisali” plant, either in terms of shoot weight or root weight, was significantly different compared with those in the “Demre” plant for both the mycorrhizal and non-mycorrhizal “Karaisali” plants under the salinity circumstances.

The mycorrhizal activity was found to be significantly more effective in the sensitive genotype. Specifically, in the “Karaisali” variety, there was a 9.8% increase in the shoot dry weight under the 75 mM salt stress, while in the “Demre” variety, this increase was 45.2% under the same conditions. The effect of mycorrhizae on the increase in the shoot dry weight under the 150 mM salt stress was recorded to be 8.9% in the “Karaisali” variety, while it was recorded to be 124.3% in the “Demre” variety, which is sensitive. Although mycorrhiza activity’s effect on the root dry weight under salt stress was lower than the rates of increase in the shoot dry weight, the AMFs were more effective on the susceptible genotype “Demre” than on the tolerant “Karaisali” genotype. At the 75 mM salt stress, the AMFs increased the root dry weight by 20% in the “Karaisali” plant and 25% in the “Demre” plant. At the 150 mM salt stress, the AMFs increased the root dry weight by 13% in the “Karaisali” genotype and 19% in the “Demre” genotype.

The implied salinity showed that the leaf chlorophyll content decreased (Table 2). The effect of AMFs on chlorophyll’s increase in the “Karaisali” genotype was 1.9% and 14.3% under 75 mM and 250 mM of salt stress, respectively. In the “Demre” genotype, under 75 mM and 250 mM of salt stress, the increasing effect of AMFs on chlorophyll was 3.7% and 7.4%, respectively.

The leaf area was not significantly affected by salt in either genotype at the beginning of the salt treatments. However, in the measurements made after 40 days of transplanting, the leaf area of the plants to which salt was applied significantly decreased. At 75 mM of salt stress, the AMFs increased the leaf area by 20.4% in the “Karaisali” plant and 14.0% in the “Demre” plant. However, at 150 mM of salt stress, the beneficial effects of AMFs on the leaf area were more pronounced, with increases of 20.5% in the “Karaisali” genotype and 50.5% in the “Demre” genotype (Table 2).

The membrane damage was lower in the tolerant genotype and greater in the sensitive genotype, but the damage was lower in the plants treated with mycorrhizae than in the plants without mycorrhizae (Table 2). The salt stress’s alleviating effect of AMFs on the cell membranes was particularly more successful at the 75 mM salt stress compared with the 150 mM salt stress. Accordingly, in the “Karaisali” genotype, the reduction in membrane damage by the AMFs was 30% and 12.8% under 75 mM and 150 mM of salt stresses, respectively. In the sensitive “Demre” genotype, the reduction rates of the membrane damage by the AMFs were 48.9% and 9.15% under 75 mM and 150 mM of salt stresses, respectively.

### 3.2. Effects of AMFs under Salt Stress on Pepper’s Physilogical Parameters

The “Karaisali” plant was able to adjust its leaf water potential by uptaking much more water via AMF contribution rather than the non-mycorrhizal “Karaisali” plant and consequently control the osmotic adjustment. Ultimately, the “Karaisali” genotype showed better performance in terms of holding water in a way to keep plants surviving in higher-salinity conditions (Table 3). Consequently, the “Karaisali” and “Demre” genotypes showed various responses to the salt concentrations in terms of leaf water potential as well as osmotic pressure. Despite neither plant having any struggles during the control treatment, as the salinity increased, significant differences in leaf water potential and osmotic potential became more visible during plant growth.

When we examined the leaf osmotic potential values, it was determined that the mycorrhiza applications improved the plants under salt stress. The leaf water potential (LWP) and leaf osmotic potential (LOP) values between the mycorrhizal and non-mycorrhizal groups with two different combinations created from “Karaisali” and “Demre” pepper plants were found to be statistically significant at the *p* ≤ 0.05 level. The best results for the LWP and LOP values, which were determined to be indicators of water status in the leaf, were obtained in the “Karaisali” control mycorrhizal applications, while the lowest values were obtained in the “Demre” non-AMF 150 mM application. The best results for the LWP and LOP values, which were determined to be indicators of the water status in the leaf, were obtained in the “Karaisali” control with mycorrhizal −1.60 MPa and −1.24 MPa applications, while the lowest values were obtained in the “Demre” non-AMF 150 mM −7.13 MPa and −2.34 MPa applications, respectively.

### 3.3. Photosynthetic Rate, Stomatal Conductance, and Transpiration Rate

The interaction between mycorrhizal fungi and plants occurred at higher photosynthesis activities, resulting in stomatal conductivity (Table 4). With a short glance at the previous results, the leaves’ chlorophyll content and size of area data could be roughly predictable in terms of having correlation with the photosynthesis rates achieved. In the meantime, the “Karaisali” plant with mycorrhizal associations performed better in terms of photosynthetic activity than the non-mycorrhizal plants. These phenomena enabled the plants to attain higher amounts of CO_2_ and H_2_O in terms of generating growth enzymes and energy for plant survival. At an initial glance, there were different photosynthetic rates between the mycorrhizal plants and non-mycorrhizal ones for both the “Karaisali” and “Demre”, plants where they were exposed to 75 and 150 mM salinity concentrations 40 days after being transplanted. The values of the photosynthetic rate almost doubled in the mycorrhizal plants compared with those in the controlled ones.

In the gas exchange parameter measurements conducted 40 days after transplantation, it was observed that the photosynthesis rate, stomatal conductance, and transpiration rate decreased in both genotypes and under both salt doses. Based on the results of the gas exchange parameters, it can be concluded that the mycorrhizae significantly supported the pepper genotypes. The application of AMFs to the pepper plants under salt stress increased their photosynthesis. The effect of the AMFs was higher at 150 mM of salt stress compared with 75 mM. The photosynthesis-enhancing effect of the AMFs was 15.6% and 51.6% under 75 mM and 150 mM of salt stress, respectively, in the “Karaisali” genotype. In the “Demre” genotype, under the same saline conditions, the rates of photosynthesis increase were 19.7% and 48.13%, respectively. The other photosynthetic parameters, namely the stomatal conductance and transpiration rate, were also consistent with the photosynthesis rates in both peppers under both salt stresses.

## 4. Discussion

The study revealed that all treatments involving mycorrhizae effectively mitigated salt stress and facilitated the water status, resulting in improved plant growth and physiological parameters.

Our study determined that mycorrhizae were more effective in terms of plant growth in sensitive genotypes under salt stress. The improvement of water relations in plants through mycorrhizal contributions to host plants is consistent with several studies [11]. Mycorrhizal fungi are confirmed to uptake up to 20% of the unavailable water captured in a plant’s rhizosphere [21]. In our study, under salt stress conditions, the pepper plants reduced their osmotic potential and increased their water uptake (Table 3). As a result, the turgor pressure increased, facilitating cell development and stomatal opening (Table 4). Studies have reported that salt stress reduces the leaf water potential in tomatoes [22,23], melons [24], and eggplants [25]. The osmotic pressure caused by high concentrations of salt and its damaging impact on plants can be significantly mitigated by the absorption of additional water through mycorrhiza hyphae during this phenomenon.

In mycorrhizal symbiosis, the fungus can mitigate or alleviate the detrimental effects of stress on the plant by inducing changes in the biochemical and physiological properties of the host plant under salt stress conditions. The mycorrhizae induce biochemical alterations in host plants under salt stress conditions, potentially resulting in increased accumulation of antioxidant enzymes, proline, betaine, or soluble carbohydrates compared with non-mycorrhizal plants. The sugar accumulation facilitated by mycorrhizae in plants serves as a defense mechanism against salinity. Elevated accumulation of soluble sugars in mycorrhiza-inoculated plants has been evidenced as a consequence of mycorrhizae enhancing photosynthesis [26,27]. In our study, the observed heightened rate of photosynthesis in mycorrhizae-inoculated pepper plants (Table 4), coupled with reduced plant damage, can be attributed to the accumulation of photosynthesis products such as sugar. This accumulation likely contributes to plant resilience against stressors. Previous studies have reported that mycorrhizal inoculation enables host plants to enhance water and nutrient uptake through hyphal networks and improve their gas exchange capacity under saline conditions [28,29]. In plants under saline conditions, mycorrhizal fungal colonization additionally (1) enhances the hydraulic conductivity of the root at low water potentials, (2) stimulates and alters the root system morphology, and (3) increases stomatal conductance. Mycorrhiza-inoculated plants exhibit high chlorophyll contents and increased N and Mg uptake, while mycorrhizae prevent Na transport in saline conditions [30,31,32,33,34,35,36,37].

Physiological changes in pepper plants inoculated with mycorrhizae compared with non-inoculated plants under salt stress include improved photosynthetic activity, stomatal conductance, and water relations (Table 4). Mycorrhizal inoculation enhances the water uptake capacity of the host plant (Table 3) by increasing hydraulic conductivity in the roots and positively affecting the osmotic balance of photosynthesis product carbohydrates, thereby contributing positively to physiological processes in the host plant. This enhancement in the water uptake capacity not only leads to increased plant growth in mycorrhizal plants but may also mitigate the toxic ion effect through dilution as the water uptake increases. Exemplary studies demonstrating this phenomenon include those by Al Karaki [38], who observed increased root dry weights and yields in salt-stressed tomatoes inoculated with mycorrhizae, resulting in higher fruit quantities and weights. Similarly, El-Sarkassy, Ibrahim, and Desoky [39] found that mycorrhizae application improved all growth parameters in pepper plants across various salt stress levels. It is known that mycorrhizal symbiosis enhances the uptake of nutrient elements and influences the phytohormone balance in plants, leading to increased plant growth and alleviation of environmental stresses. Consequently, it promotes biomass and yields in plants and has a positive effect on some quality parameters [40,41,42]. Under salt stress, photosynthesis decreases in plants. This may be attributed to disruption of the water balance, alteration of the enzyme structure, and inhibition of protein synthesis by specific ions (e.g., Na+) [43]. In our study, we observed higher plant growth parameters (Table 1 and Table 2) and higher photosynthetic properties in plants treated with mycorrhizae (Table 4).

In salt stress conditions, reduced stomatal conductance [44] and enzyme inactivation [45] result in CO_2_ accumulation among cells. Elevation in the intercellular CO_2_ concentration indirectly suggests damage to photosynthetic organs [27]. Zhang et al. demonstrated in their study on tomatoes that mycorrhizal inoculation increased photosynthesis by enhancing CO_2_ assimilation in tomato plants through non-stomatal factors [46]. Mycorrhizae significantly elevated the concentration of photosynthetic pigments in tomato leaves grown in saline-alkaline soil, thereby facilitating the capture and utilization of light energy. As is known, the efficiency of plant photosynthesis is closely linked to growth and development. According to the findings of our study, the photosynthesis rate was higher in pepper plants treated with mycorrhizae, leading to increased leaf areas and shoot dry weights. A similar study demonstrated that mycorrhizal symbiosis enhances photosynthesis in plants, leading to increased assimilate accumulation, as well as improved mineral nutrition and fruit quality [47].

In this study, mycorrhizal symbiosis altered the plants’ physiological aspects, such as water relations in leaves, consequently enhancing photosynthetic activities in both the “Karaisali” and “Demre” plants subjected to salinity stress. These results suggest that the association (symbiosis) of both plants with mycorrhizal fungi may improve growth capability by increasing water uptake through external hyphae and enhancing the osmotic potential through the provision of salt and high concentrations of minerals.

## 5. Conclusions

Climate change, which is increasingly evident worldwide, necessitates cultivation in saline soils due to improper soil management practices. While the use of salt-tolerant varieties seems to be an immediate solution, a more sustainable approach involves harnessing nature-friendly beneficial microorganisms like mycorrhizal fungi to cultivate productive and high-quality, salt-sensitive varieties and mitigate salt-induced plant damage. Moreover, the dwindling availability of fertilizer resources and rising production costs forecast serious efficiency challenges in crop production over the next 50 years. Therefore, leveraging mycorrhizal fungi, which reduces reliance on chemical fertilizers, proves ecologically sound and vital for agricultural sustainability. Recognizing the potential of mycorrhizae as a natural fertilizer in saline soils for promoting mycorrhizal inoculation in mycorrhizae-dependent plants has emerged as an increasingly crucial agricultural strategy. In this study, it was shown that the growth, water status, and photosynthesis characteristics of the “Demre” salt-sensitive pepper genotype were significantly improved by AMFs at medium and high salinity levels such as 75 mM and 150 mM NaCl, respectively.

## Figures and Tables

**Table 1:** “Karaisali” and “Demre” biomass productions, including shoot and root dry weights, during different salinity concentrations with and without AMF inoculation 40 days after transplanting and after 26 days under salinity (g plant^−1^).

Variety	Mycorrhyzae	Salt Doses	Shoot Dry Weight	Root Dry Weight
“Karaisali” salt-tolerant	M (+)	0 (control)	9.34 a	2.18 a
		75 mM	5.59 b	1.26 a
		150 mM	3.30 b	0.67 c
	M (−)	0 (control)	8.58 a	2.16 a
		75 mM	5.09 b	1.05 b
		150 mM	3.03 b	0.59 c
“Demre” salt-sensitive	M (+)	0 (control)	7.73 a	1.35 a
		75 mM	4.43 bc	0.75 b
		150 mM	3.05 cd	0.50 b
	M (−)	0 (control)	6.74 ab	1.24 b
		75 mM	3.05 cd	0.60 b
		150 mM	1.36 d	0.42 b
*p* ≤ 0.05			0.152	0.067

Different letters within a column indicate significant differences at *p* ≤ 0.05.

**Table 2:** Comparison of mycorrhizal association’s effects on leaf chlorophyll content, leaf area, and membrane damage in both mycorrhizal and non-mycorrhizal plants of the two pepper genotypes under different saline concentrations 40 days after transplanting and after 26 days under salinity.

Variety	Mycorrhyzae	Salt Doses	Chlorophyll (SPAD)	Leaf Area (cm^2^ plant^−1^)	Membran Injury Index (%)
“Karaisali” salt-tolerant	M (+)	0 (control)	72.10 a	837.93 a	
		75 mM	68.60 a	605.48 ab	2.64
		150 mM	67.33 a	491.04 b	5.87
	M (−)	0 (control)	71.93 a	837.93 a	
		75 mM	67.33 a	502.86 b	3.77
		150 mM	58.93 b	407.61 b	6.73
“Demre” salt-sensitive	M (+)	0 (control)	69.30 a	761.68 a	
		75 mM	64.30 ab	579.89 ab	2.92
		150 mM	57.03 bc	340.10 ab	10.62
	M (−)	0 (control)	70.23 a	805.09 a	
		75 mM	62.03 ab	508.56 ab	5.71
		150 mM	53.10 c	226.01 b	11.69
*p* ≤ 0.05			0.199	0.069	0.094

Different letters within a column indicate significant differences at *p* ≤ 0.05.

**Table 3:** Comparison of mycorrhizal association effects on leaf water potential and osmotic potential in both mycorrhizal and non-mycorrhizal plants of two pepper genotypes under different saline concentrations 40 days after transplanting and after 26 days under salinity.

Variety	Mycorrhyzae	Salt Doses	Leaf Water Potential (MPa)	Osmotic Potential (MPa)
“Karaisali” salt-tolerant	M (+)	0 (control)	−1.60 f	−1.24 e
		75 mM	−3.47 d	−1.70 b
		150 mM	−5.13 bc	−1.87 ab
	M (−)	0 (control)	−2.07 e	−1.33 d
		75 mM	−3.90 cd	−1.77 b
		150 mM	−5.73 bc	−1.96 ab
“Demre” salt-sensitive	M (+)	0 (control)	−2.27 de	−1.31 d
		75 mM	−4.40 c	−1.85 ab
		150 mM	−6.07 b	−2.03 ab
	M (−)	0 (control)	−3.67 d	−1.65 c
		75 mM	−4.70 c	−1.98 ab
		150 mM	−7.13 a	−2.34 a
*p* ≤ 0.05			0.001	0.367

Different letters within a column indicate significant differences at *p* ≤ 0.05.

**Table 4:** Comparison of mycorrhizal association effects on photosynthetic and gas exchange parameters in both mycorrhizal and non-mycorrhizal plants of two pepper genotypes under different saline concentrations 40 days after transplanting and after 26 days under salinity.

Variety	Mycorrhyzae	Salt Doses	Photosynthetic Rate (μmol CO_2_ m^−2^s^−1^)	Stomatal Conductance (mmol H_2_O m^−2^ s^−1^)	Transpiration Rate (mmol H_2_O m m^−2^ s^−1^)
“Karaisali” salt-tolerant	M (+)	0 (control)	9.94 a	0.13 a	1.71 a
		75 mM	8.53 ab	0.07 ab	1.21 ab
		150 mM	5.20 cd	0.04 b	0.66 c
	M (−)	0 (control)	9.65 a	0.11 a	1.57 a
		75 mM	7.38 b	0.06 ab	0.85 b
		150 mM	3.43 d	0.03 c	0.50 cd
“Demre” salt-sensitive	M (+)	0 (control)	8.71 ab	0.09 a	1.30 ab
		75 mM	6.61 bc	0.06 ab	1.04 ab
		150 mM	3.97 d	0.03 c	0.44 cd
	M (−)	0 (control)	7.71 ab	0.07 ab	1.29 ab
		75 mM	5.52 c	0.03 b	0.56 c
		150 mM	2.68 e	0.02 c	1.71 a
*p* ≤ 0.05			0.047	0.011	0.020

Different letters within a column indicate significant differences at *p* ≤ 0.05.

## Data Availability

Data are contained within the article.
